# Guided Growth Technique for Epiphysiodesis and Hemiepiphysiodesis: Safety and Performance Evaluation

**DOI:** 10.3390/children11010049

**Published:** 2023-12-29

**Authors:** Giovanni Luigi Di Gennaro, Giovanni Trisolino, Stefano Stallone, Marco Ramella, Gino Rocca, Giovanni Gallone

**Affiliations:** 1 Unit of Pediatric Orthopedics and Traumatology, IRCCS Istituto Ortopedico Rizzoli, 40136 Bologna, Italy; giovanniluigi.digennaro@ior.it (G.L.D.G.); giovanni.trisolino@ior.it (G.T.); marco.ramella@ior.it (M.R.); gino.rocca@ior.it (G.R.); 2 Unit of Orthopedics and Traumatology, Ospedale Maggiore “Pizzardi”, 40133 Bologna, Italy; stefano.stallone@ausl.bologna.it

**Keywords:** guided-growth, angular deformity, limb-length discrepancy, 8-Plate Plus, Guided-growth Plate System Plus, GGPSP, Orthofix

## Abstract

Background: Guided-growth modulation is a first-line treatment widely adopted to correct lower-limb angular deformities and limb-length discrepancies (LLD) in the paediatric population. Methods: We conducted a retrospective study to evaluate the safety and performance of a new construct (8-Plate Plus or Guided-Growth Plate System Plus, Orthofix S.r.l) used to correct angular deformities and LLD in non-skeletally mature children. The primary endpoint was safety (from plate implantation to removal). The secondary endpoint was performance; patients treated for LLD achieved complete correction if a pre- and post-surgery difference of <0 was observed; angular deformities performance was measured in terms of IMD, ICD, mMPTA, and mLDFA. Results: We performed 69 procedures in 41 patients. A total of 10 patients had an LLD, and 31 had an angular deformity. We observed nine minor complications in the hemiepiphysiodesis group. One patient experienced rebound. All 10 LLD patient treatments were successful. A total of 30/31 patients with an angular deformity had a successful treatment; the remaining patient had a partial correction. Conclusions: Guided-growth by temporary epiphysiodesis or hemiepiphysiodesis was safe and effective for angular deformities and limb-length discrepancies. Further prospective and/or randomized controlled trial studies assessing more significant cohorts of patients and a comparison group could add evidence to our findings.

## 1. Introduction

Paediatric orthopaedists state that lower-limb angular deformities are one of the most frequently non-traumatic conditions being researched [1,2,3]. While lower limb position tends to change during growth, from slight varus to slight valgus between 1 and 7 years of age, past this threshold, a paediatric subject is considered to have a neutral alignment [3,4,5]. When this is not the case, lower-limb deformities may compromise gait, produce pain, give instability, and limit functionality [3,4]. Lower-limb deformities may include both idiopathic and pathological malalignment and limb-length discrepancies. Obesity is known to be a risk factor, but other factors may not be that predictable [5].

Guided growth or modulation of the growing bone is widely approved as the first-line approach when conservative treatment has failed [4,6]. This is mainly because it is simple to technically assimilate and perform, is minimally invasive and reversible, gives rise to lesser evident scars, and has fewer complications compared to other more invasive treatments such as osteotomy with acute correction and stabilization, physeal bar resection, or multi-planar dynamic external fixation [3,4,6].

Although these procedures are deemed to be safe and effective, proper indication and meticulous application of the standard technique are essential to good outcomes [4]. Timely intervention is paramount for a successful outcome, as late indication may lead to incomplete corrections [3,4,7]. The treatment aims to restore limb alignment, arrest progression, and prevent recurrence after the intervention [6,8].

Guided-growth plating has shown positive results in different settings, from multiple osteochondromas to idiopathic cases [7].

There are several 8-shaped plates on the market. The Guided-Growth Plate System Plus (GGPSP) produced by Orthofix, Bussolengo, Italy—also known as 8-Plate Plus and released in 2021—is a new construct that differs from its predecessor, the Guided-Growth System or 8-Plate, in the dimensional range of the implantable components (plates and screws). The new construct also allows for a higher degree of motion, which may provide a better outcome in patients needing to stay on the plate longer and might generate fewer device-related complications. In particular, the total height of the system (screw plus plate) is inferior to the old version (3.5 mm vs. 4.1 mm) in the early post-operative construct, adding a “low profile” feature that could be linked to better patient tolerance. Furthermore, the screws have a higher time for reaching maximum angulation. This arrangement, where the total height of the system is 4.5 mm, like in the first generation, could lead to a better outcome in terms of daily pain. To our knowledge, this study is the first one reporting clinical data on this new device. It aims to evaluate the safety and performance of its use in non-skeletally mature children diagnosed with a lower-limb angular deformity or leg-length discrepancy.

## 2. Materials and Methods

### 2.1. Study Design and Participants

We conducted a retrospective study on non-skeletally mature children with deformities of femur and tibia (most frequent sites of applications), admitted at our institution—a national referral centre for rare skeletal disorders (Rizzoli Orthopedic Institute, Bologna, Italy).

Patients were eligible if they (1) had been treated with an 8-Plate Plus to correct deformities of the femur and/or tibia; (2) were not skeletally mature (according to the pre-surgical X-ray, the growth plates of the treated limb(s) were still evident); (3) had completed therapy with 8-Plate Plus and had already attended at least one post-plate removal check-up; (4) had an accessible dataset for the assessment of safety and clinical efficacy of treatment.

On the contrary, patients were not eligible if they (1) had been treated with an 8-Plate Plus for an off-label anatomical location; (2) had been treated with concomitant devices other than the 8-Plate Plus (unless these did not compromise the safety and performance of the primary treatment); (3) were still being treated with 8-Plate Plus or had not attended any post-plate removal check-ups; (4) did not have an accessible dataset for the assessment of safety and the clinical efficacy of treatment.

The institutional review board approved the study protocol (ID: 663/2021/Oss/IOR—n. 0012656) according to Italian regulations (art. 110bis of decree n.196/2003). Explicit consent was not required for retrospective chart reviews and publication of the aggregated data. The study followed the Declaration of Helsinki (Fortaleza, October 2013) and the Good Clinical Practice (ICH-GCP) guidelines.

### 2.2. Surgical Procedure

The surgical procedure, conducted as part of the hospital’s routine practice, was planned and performed by a team of 11 paediatric orthopaedists, following the standard treatment at our institution.

Under general or local anaesthesia, the patient was placed, as standard technique required, in supine position on a radiolucent operating table with a thigh tourniquet. Using fluoroscopy, femoral and/or tibial physes were exhibited, the skin marked and incised about 2–3 cm from the level of the identified area and dissected down to the periosteum.

8-Plate Plus (Guided-Growth Plate System Plus, Orthofix Srl, Bussolengo, Italy) was placed, centred, over the growth plates. Implant size was chosen according to the size of the knee. Sutures of the fascia and subcutaneous planes with final closure of the skin using running absorbable wire were then performed.

After surgery, children could fully move around and walk with full weight bearing and were usually discharged by the second or third day after the intervention. Clinical assessments were conducted at regular intervals post-operatively at one and three months to check the status of the deformity correction. The plate was removed once the corrected alignment was reached or after a slight overcorrection following the surgeon’s post-operative planning.

### 2.3. Assessment of Baseline Variables and Outcomes

At baseline, demographic data included race, gender, and age, while anthropometric parameters including body weight, height, body mass index (BMI), and related z-scores were evaluated according to the CDC calculator (https://www.cdc.gov/healthyweight/bmi/calculator.html, accessed on 1 December 2021).

Radiographic parameters at baseline, obtained from standard standing X-rays that involve the segments from pelvis to foot (standard long leg view) included mechanical hip–knee–ankle angle (HKA) and mechanical axis of deviation (MAD); anatomical lateral distal femoral angle (aLDFA) and mechanical distal lateral femoral angle (mLDFA); mechanical medial proximal tibial angle (mMPTA); distal lateral tibial angle (LDTA); the tibia–fibula ratio (tibial length/fibular length), and femur–tibia ratio (femur length/tibial length) [9,10].

The severity of the angular deformity of the knee was evaluated using the intermalleolar distance (IMD) and intercondylar distance (ICD) scores [5,11,12].

During and after surgery, the following parameters were recorded: age at the time of surgery; surgery details (duration, complications, post-operative X-rays) and data on the device(s) implanted (i.e., quantity, type, and size); medications and concomitant treatments; treatment time (from plate implantation to removal).

We measured safety endpoints as expected, and unexpected serious adverse events and hardware complications (breakage, bending, etc.) were recorded from plate implantation to removal. Once the device was removed from the application site, whether the correction had been maintained was evaluated during the post-plate removal visit. The objective was to verify whether rebound cases, typical of these conditions, had occurred.

**Figure 1:**
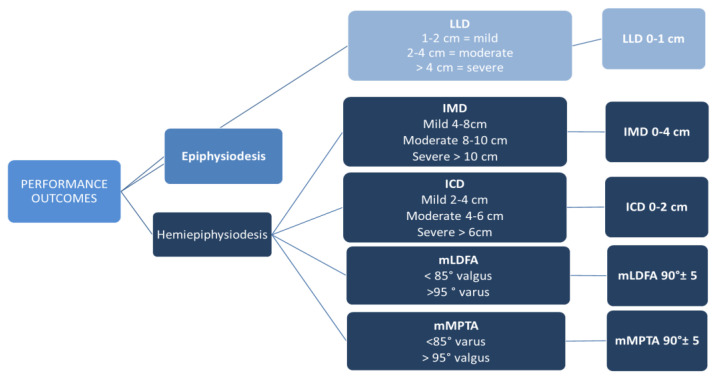
Performance outcome parameters and definition of complete correction. mMPTA: mechanical medial proximal tibial angle; mLDFA: mechanical lateral distal femoral angle; IMC: intermalleolar distance; ICD: intercondylar distance.

Performance endpoints were recorded at plate removal and six months past plate removal. Performance was measured in terms of complete correction (full response to treatment). Performance parameters are shown in Figure 1.

### 2.4. Sample Size Estimation

Because the growth-plate technique is a consolidated treatment and its performance widely proven in the scientific literature, the safety of this reversible treatment was considered for the calculation of the sample size.

The scientific literature reports that the percentage of patients treated with a tensioning plate for a lower-limb deformity who faced at least one device-related complication was between 3% and 12%, depending on many factors, mainly the heterogeneity of the prevalent cause [13]. Assuming that the expected percentage of patients with at least one complication for the 8 Plate Plus was to be 3%, the sample size calculation showed that the upper limit of the 95% confidence interval for the primary endpoint based on the exact binomial method must remain below 12 per cent (i.e., 9.94%); thus, 70 procedures (performed on approximately 40 patients) were needed.

### 2.5. Statistical Analysis

The clinical data were analysed using descriptive statistics: the primary endpoint is presented as a percentage value and the corresponding 95% confidence interval; the secondary endpoint is presented as a percentage value and the corresponding 95% confidence interval.

## 3. Results

### 3.1. Patients’ Characteristics at Baseline

Between January 2019 to September 2020, 141 children underwent guided growth by temporary hemiepiphysiodesis or epiphysiodesis. Of them, 42 children (with 70 knees) were treated using the 8-Plate. One patient was lost to follow up, leaving 41 children (69 knees) available for the analysis. The mean age at surgery was 12.3 ± 1 years, and the overall treatment time was 618.5 days (499.0–928.0). Patients’ characteristics at baseline are reported in Table 1.

A total of 10 patients were obese and 27 patients had comorbidities. Some patients presented more than one comorbidity. See Table 2.

### 3.2. Surgery

The procedure was carried out in 24 patients (58.5%) under local anaesthesia and in 6 (14.6%) cases under general anaesthesia; in the remaining patients, other types of anaesthesia were used (infiltration + local, sedation + local, total + local).

We used one plate in 59 knees that underwent hemiepiphysiodesis (28 bilateral) (85.5%), and 10 cases received epiphysiodesis (14.5%). The plate application site was predominantly in the distal femoral region in 57 knees (82.65%), while, in 12 (17.4%), the application was carried out in the proximal tibial region.

In nine patients, in addition to the 8-Plate Plus, a screw was applied to the patient’s heel to correct flat foot. One patient sustained combined intramedullary nailing to treat an impending fracture of the femur due to monostotic fibrous dysplasia.

In 40 patients, antibiotic and anti-inflammatory treatments were administered in the post-surgery phase following standard clinical practice.

Surgical data are reported in Table 3.

### 3.3. Safety

No complications occurred during surgery.

Nine minor complications were recorded in the hemiepiphysiodesis group during follow-up. Detailed information is presented in Table 4. None of the epiphysiodesis cases experienced any complication. All complications required just some minimal treatment, like painkillers, physiotherapy, oral antibiotics (for pain, stiffness, and superficial infection), or none, and they were deemed as not clinically relevant (Grade I Modified Clavien–Dindo–Sink Classification). No complications regarding the device used, like screw breakage, failure of correction, screw pull-out, screw/plate migration, growth disturbance, or physeal injury, were experienced.

### 3.4. Performance

Regarding the evaluation of the clinical benefit of treatment, the 69 procedures included in the study were divided as follows:

− 10 (14.5%) cases of epiphysiodesis in 10 patients
− 59 (85.5%) cases of hemiepiphysiodesis in 31 patients

One patient did not maintain the degree of correction (the only case of rebound complication) due to the increase in weight and thigh fat, which caused a rise in IMD compared to the previous visit.

#### 3.4.1. Epiphysiodesis

Ten patients were treated with epiphysiodesis for LLD. All of them were classified as “low” or “moderate” LLD (from 1 to 4 cm). There were not “severe” LLD cases due to our current clinical practice of favouring a limb lengthening in these cases to avoid a final short height.

The median duration of treatment (calculated as the interval between the application of the plate and its removal) was 656.8 days (range: 499 to 928 days).

In seven patients, the LLD was of the femur, while, in three patients, it was of the tibia. In subject 7, the procedure was carried out in the femur, even if the tibia was the bone segment in question, due to pathological growth plates (Metachondromatosis/Ollier disease). At final follow-up, the complete (femur + tibia) LLD was nullified. All the patients treated reached complete correction (as the performance outcome stated, LLD lower than 1 cm). The mean correction rate for distal femur epiphysiodesis was 1.43 (SD 0.82) mm/month, and it was 0.75 (SD 0.35) mm/month for proximal tibia. See Table 5.

#### 3.4.2. Hemiepiphysiodesis

Of the 41 patients included in the study, 31 were treated with hemiepiphysiodesis, corresponding to 59 procedures.

The median duration of treatment (calculated as the interval between the application of the plate and its removal) was 453.8 days (range: 241 to 774 days).

For all 59 procedures (31 patients), pre- and post-operative mLDFA and mMPTA values were recorded. For the 49 treatments of valgus deformity at the distal femur, the mean mLDFA increased from 85.3° ± 3.1° (range, 78° to 91°) pre-operatively to 93.2° ± 4.2° (range, 86° to 103°) post-operatively, with a mean angle variation of 7.9°. In these cases, the mean rate of mLDFA angle correction was 0.57°/month. For the three treatments of valgus deformity at the proximal tibia, the mean mMPTA decreased from 97.3° ± 5.1° (range, 93° to 103°) pre-operatively to 87.7° ± 1.5° (range, 86° to 89°) post-operatively, with a mean angle variation of −9.7°. In these cases, the mean rate of mMPTA angle correction was −0.14°/month. For the seven treatments of varus deformity at the proximal tibia, the mean mMPTA increased from 84.1° ± 3.8° (range, 79° to 88°) pre-operatively to 90.4° ± 3.9° (range, 84° to 97°) post-operatively, with a mean angle variation of 6.3°. In these cases, the mean rate of mMPTA angle correction was 0.09°/month. Data are summarized in the table below (Table 6).

Radiological parameters were not fully obtained at the final follow-up because, considering the retrospective nature of the study, X-rays were not deemed necessary in the outpatient clinic visit. In our current clinical practice, we tend to avoid radiological hazards if they are not indispensable, especially on paediatric patients; for that reason, we used IMD and ICD as indicators for possible rebound effect, even though we acknowledge that they could not be as precise as radiological standards.

Out of a total of 31 patients, in 6 patients (12 knees), it was not possible to evaluate the intermalleolar or intercondylar distance (IMD or ICD) and, therefore, for the evaluation, mLDFA and mMPTA parameters were used to assess the difference before and after application of the plates. A total of 11 knees (91.7%) achieved complete correction, according to the criteria for the femoral or tibial angle variation. One knee (8.3%) did not achieve correction. Nevertheless, according to the investigator’s opinion, the case had reached partial or complete correction, as there had been an improvement in stature and gait at the time of the clinical evaluation, probably due to weight loss.

One patient was evaluated with both criteria and achieved a full correction as expected.

In the remaining 24 patients (77.4%), it was possible to evaluate the intermalleolar or intercondylar distance (IMD or ICD), and the change in score before and after application of the plate(s) was therefore assessed. All 24 achieved a full correction with a significant change in deformation (100%). See Table 6.

## 4. Discussion

This observational retrospective study analysed a cohort of patients undergoing guided growth with an 8-Plate Plus to correct genu valgus, genu varus, or limb-length discrepancy.

Timely intervention is paramount for a successful outcome [14], as late or incorrect indication may lead to incomplete corrections, risk of secondary deformities, and rebound effect, as several authors have consistently stated [3,4,7]. The precise timing is really the main aspect to fully understand in order to accomplish a satisfactory outcome and eliminate or minimize the risk of complications. For example, in our current practice, growth-plate modulation is advised in pre-adolescent subjects, regarding typically idiopathic cases, to exploit the puberal spurt and achieve correction in relatively lesser time. Also, in our cohort, the average age at surgery was 12.3 years old. In the case of males, delaying intervention may be possible, whereas, in the case of females, it is generally advisable to intervene at an earlier stage, as bone age correlates with chronological age for both sexes at the beginning of puberty but not at the end [15]. Our patient population is comparable to that of previous and recent studies [16,17,18,19,20]. Besides, if the patient has associated pathologies, it is also advisable to intervene at an earlier stage. This is probably connected to a not fully functional growth plate, differently from what is seen in the idiopathic cases. Furthermore, the increased risk of relative shorter height in these kinds of conditions could be another factor in favour of forerunning the surgical treatment [7].

All our patients treated for LLD of the femur or tibia with this new construct (8-Plate Plus or GGPSP) reached full correction. Borbas and colleagues published similar results using a previous version of the GGPSP (the Guided-Growth Plate, manufactured by Orthofix Srl., Bussolengo Verona, Italy) in 2019 [17], after a study comparing definitive percutaneous epiphysiodesis versus temporary epiphysiodesis with this device. The reduction of the LLD in 12 months was 5.7 mm in patients treated with the tension band plate and 8.4 mm with definitive treatment. This difference was, however, statistically not significant (38 patients were included overall, which does not allow for clear significant analysis). Demirel et al. [18] reached limb-length discrepancy correction in 54.5% of the patients studied (11 patients) using a different 8-shaped plate.

Regarding angular correction, our study shows that this technique is safe and effective in correcting angular deformities during growth, achieving 97.6% (40 out of 41 patients) success. Similar results with different guided-growth plates have been reported by Park and colleagues [19]: 97.1% of patients achieved full corrections (a retrospective study including 35 patients treated with the R-plate). But, previous studies did not report such encouraging results: Quintero and colleagues [20] reported an 81.7% achievement of correction on 115 plates; Ellsworth et al. [21] reported 82.4% on 17 patients; and Ghaznavi and colleagues [22] reported 95.45% on 109 patients. In the end, in terms of safety and effectiveness, our results on the GGPSP are comparable to the recent literature regarding guided-growth techniques using 8-shape plates. This kind of construct is currently one of the most (if not the most) used system for temporary hemiepiphysiodesis/epiphysiodesis. For this reason, future studies with larger series may disclose possible advantages in using 8-Plate Plus in comparison to other 8-shape plates.

The guided-growth technique is easy to perform and is not influenced by the surgeons’ experience, as seen from the present study, in which a team of 11 different surgeons, with different years of experience, operated on the whole sample. And, overall, the body of literature suggests that it is an advisable first approach, given the fact that it is reversible [17,18,19,20,21,22,23,24].

Limitations of the current study include the small number of patients, which may not provide a statistically significant result and may have limited power to detect an actual effect due to the small sample size. It also increases the likelihood of chance effects or errors in the results. Limitations also include the study’s observational nature, which lacked a comparator. Thus, the effect of treatment cannot be attributed solely to the intervention. Finally, our study was conducted in a single centre and may not be representative of other settings or populations. That is why broader multicentre studies regarding this matter could add important evidence in terms of safety and performance in wide and varied populations. The relative simplicity of the surgical technique and expansive indication from idiopathic to pathological deformities could be helpful for reaching strong results.

With the emerging technologies of assisted pre-planning with software and 3D printing, the field of guided-growth systems is expected to see significant developments in the future. These technologies allow doctors to create a customised treatment plan for each patient based on individual needs and unique anatomical considerations [23]. These systems are already used with alternative yet more invasive treatment approaches.

## 5. Conclusions

In conclusion, guided growth by temporary epiphysiodesis or hemiepiphysiodesis was safe and effective in correcting femoral and tibial axial deformities and limb-length discrepancy in idiopathic and pathologic children, with results visible after 6 months past the removal of the plates. Further prospective and/or randomized control trial studies assessing bigger cohorts of patients and with a comparison group could add evidence to our findings.

## Figures and Tables

**Table 1:** Patients’ characteristics at baseline.

Variables		
Per patient data		
Gender, n (%)		
Female		15 (36.5)
Male		26 (63.4)
Age, years	Mean (SD)	12.3 (1.04)
Height, cm	Mean (SD)	155.0 (10.05)
Weight, kg	Mean (SD)	55.3 (12.00)
BMI	Mean (SD)	22.9 (3.76)
Right Femur LLD	n	5
Left Femur LLD	n	3
Right Tibia LLD	n	1
Left Tibia LLD	n	1
Right Knee Valgus	n	1
Left Knee Valgus	n	1
Right Knee Varus	n	1 25
Right Knee Valgus/Left Knee Valgus Right Knee Varus/Left Knee Varus Per procedure data	n	3
HKA	n (average)	69 (0.80)
MAD	n (average)	69 (3.3)
Severity of IMDc		
Moderate (8 < IMD/ICD < 10 cm)	n (%)	7 (11.9)
Severe (IMD or ICD > 10 cm)	n (%)	40 (67.8)

n: number; SD: standard deviation; IMD: intermalleolar distance; ICD: intercondylar distance; HKA: hip–knee–ankle angle; MAD: mechanical axis deviation. Note: IMC was not evaluable in 10 cases, and information was missing in 2 cases.

**Table 2:** Patients’ comorbidities.

Variables	Angular Deformity pts—n	Limb-Length Discrepancy pts—n	Total n (%)
Multiple exostosis disease, n (%)	4	1	5 (12.75)
Sequelae of tibia osteosynthesis surgery for fracture	1	0	1 (1.44)
Calcaneal stop screw for correction of a valgus deformity of the back foot (subtalar joint)	10	3	13 (18.84)
Sequelae of right hip Perthes’ disease	0	1	1 (1.44)
Hypoplasia of trapezius muscle and left scapula elevators	2	0	2 (2.89)
Tetralogy of Fallot, hand agenesia	0	1	1 (1.44)
Hypostaturism, previous epiphysiodesis intervention for knee valgus deformity	2	0	2 (2.89)
Metachondromatosis/Ollier’s disease	1	0	1 (1.44)
Fracture of the tibia (8 years earlier)	0	1	1 (1.44)
Femoral fracture (at 2.5 years of age)	0	1	1 (1.44)
Monostotic fibrous dysplasia treated with splint	1	0	1 (1.44)

n: number.

**Table 3:** Surgical data considering procedures.

Variables		
N. of plates, n (%)		
1—hemiepiphysiodesis		59 (85.5)
2—epiphysiodesis		10 (14.5)
Plate dimension, n (%)		
12 mm		14 (20.3)
16 mm		55 (79.7)
Screws, n (%)		
Cannulated 24 mm		52 (75.36)
Cannulated 32 mm		17 (24.63)
N. of screws, n (%)		
2		59 (85.5)
4		10 (14.5)
Duration of surgery, median in min (min/max)		27 (15/53)

**Table 4:** Complications per patient.

Variables	
Superficial infection, n (%)	1 (2.43)
Pain, n (%)	2 (4.87)
Stiffness, n (%)	1 (2.43)
Keloid scarring, n (%)	1 (2.43)
Hypertrophic scar, n (%)	1 (2.43)
Mild medial swelling, n (%)	1 (2.43)
Slight limitation of knee extension, n (%) Wound bleeding	1 (2.43) 1 (2.43)
Rebound	1 (2.43)

n: number.

**Table 5:** Results of the cases treated for LLD.

Variables (Case, Diagnosis)	Pre-Surgery			Post-Surgery		
	Healthy Limb (cm)	Operated Limb (cm)	LLD (cm)	Healthy Limb (cm)	Operated Limb (cm)	LLD (cm)
1. Right Femur LLD	42	43	−1	44	43	1
2. Left Femur LLD	46	47	−1	51	50	1
3. Right Femur LLD	53	54	−1	56	56	0
4. Left Femur LLD	45	46	−1	52	52	0
5. Right Femur LLD	46	48	−2	50	50	0
6. Right Femur LLD	48	49	−1	55	55	0
7. Left Femur LLD	45	45	0	50	48	2
8. Right Femur LLD	42	44	−2	48	48	0
9. Right Tibia LLD	31	33	−2	33	34	−1
10. Left Tibia LLD	39	41	−2	42	43	−1

**Table 6:** Evaluation of the improvement of hemiepiphysiodesis: pre- and post-surgery mean values through the assessment of mLDFA and mMPTA angles and severity of the IMD or the ICD.

		**mLDFA**			**mMPTA**		
**Deformity**	**Anatomical Site, n of Cases**	**Pre-Surgery** **Angle (°)** **Mean ± SD**	**Post-Surgery** **Angle (°)** **Mean ± SD**	**Angle** **Variation (°)** **Mean**	**Pre-Surgery** **Angle (°)** **Mean ± SD**	**Post-Surgery** **Angle (°)** **Mean ± SD**	**Angle** **Variation (°)** **Mean**
Valgus	Distal femur, 49	85.3 ± 3.1	93.2 ± 4.2	7.9			
	Proximal tibia, 3				97.3 ± 5.1	87.7 ± 1.5	−9.7
Varus	Proximal tibia, 7				84.1 ± 3.8	90.4 ± 3.9	6.3
**Intermalleolar Distance (IMD)** **Intercondylar distance (ICD)**			**No. of** **Patients Pre-Surgery**	**%**	**No. of** **Patients at Final** **Follow-Up**	**%**	
Normal (IMD or ICD <4 cm)			0	0.0	20	80	Yes
Mild (4 cm < IMD or ICD <8 cm			0	0.0	5	20	Yes
Moderate (8 cm < IMD or ICD <10 cm			4	16	0	0.0	-
Severe (IMD or ICD >10 cm)			21	84	0	0.0	-

## Data Availability

Data available on request due to restrictions. The data presented in this study could be available on request from the corresponding author. The data are not publicly available due to national privacy regulations.
